# Very Small Home Ranges of Two Gravid European Brown Bears during Hyperphagia

**DOI:** 10.3390/ani11123580

**Published:** 2021-12-17

**Authors:** Laura Schulte, Daniele De Angelis, Natarsha Babic, Slaven Reljić

**Affiliations:** 1 Department of Behavioural Ecology, Bielefeld University, 33615 Bielefeld, Germany; 2 Department of Biology and Biotechnology “Charles Darwin” BBCD, Sapienza University of Rome, 00185 Rome, Italy; deangelis.daniele@yahoo.it; 3 School of Biological Sciences, Clayton Campus, Monash University, Melbourne, VIC 3800, Australia; natarsha.babic1@monash.edu; 4 Biology Department, Faculty of Veterinary Medicine, University of Zagreb, 10000 Zagreb, Croatia; slaven.reljic@gmail.com

**Keywords:** seasonal home range, Brownian Bridge Movement Model, GIS analyses, *Ursus arctos*, GPS-telemetry, gravidity, Paklenica National Park, Velebit Mountains, Croatia

## Abstract

**Simple Summary:**

Paklenica National Park is home to the European brown bear while it is also frequently visited by tourists and home to permanent and semi-permanent residents. The aim of our study was to analyze the use of space of the National Park in autumn. Therefore, we have live captured two brown bears in September 2019 and equipped them with GPS/GSM collars to track their movement pattern and then estimate their home range. We captured two females that were both gravid. We found out that these individuals used very small seasonal home ranges in autumn before denning. Additionally, they almost exclusively showed solitary use of their home range. They nevertheless spent a considerable amount of time close to feeding sites and approached human settlements as close as 4 m while they were mostly active during the night. During the pre-denning stage, most human–bear encounters occur, which is why it is important to offer refugia for the animals from human disturbance.

**Abstract:**

In September 2019, two gravid female brown bears (*Ursus arctos*) were captured and equipped with GPS/GSM collars in Paklenica National Park (Croatia). Home ranges during hyperphagia were analyzed to describe the spatiotemporal requirements. Mean seasonal home ranges were very small with 9.2 km^2^ and 7.5 km^2^ (Brownian Bridge Movement Model 95%). During the tracking period, both bears used different territories and showed little to no use of overlapping area. The bears in our study spent a considerable time in proximity of artificial feeding sites, indicating a probable use of these structures as a food resource (mean 15.7% and 30.7%). Furthermore, the bears approached very close to human structures such as 8.9 m and 4.4 m. As most encounters between humans and bears occur during hyperphagia, it is important to offer refugia from human disturbance, especially as the National Park is not only used by residents, but also by tourists. To adapt management according to the animal’s needs, further studies should include more individuals from different age and sex classes. Both females were gravid. It remains unclear whether gravidity has an effect on the home range and should be further investigated.

## 1. Introduction

Conservation of species in times of changing environmental conditions and continuously rising anthropogenic exploitation of natural resources and land use is probably one of the most crucial challenges wildlife management must face. This becomes even more perceptible in conservation of large carnivore species [1,2,3] because they have small population sizes, low fecundity rates, larges spatial requirements and occupy high trophic levels [4]. The brown bear (*Ursus arctos*), as one of the large carnivore species of Europe [5], has only recently increased its numbers in the Dinaric Mountains and Croatia due to conservation efforts after declining over the past few decades [6,7]. Several factors can influence the home range and habitat choice of brown bears, but none is as delicate as human–bear interactions [8,9]. Human–bear conflicts increase with bear population recovering in Europe as humans are extending their activities towards nature use and bears can cause damage to livestock, agriculture, and orchards [10,11], whereas most human–bear encounters occur in the hyperphagic period in autumn as reported from Iran [10]. In general, the behavior of European brown bears is known to adapt to human presence, which is, for instance, expressed through a shift in activity pattern and spatial use [8]. This applies to the use of their natural habitat as well as human structures. Therefore, human activity needs to be considered as an important influencing factor [2,4,12,13].

Besides anthropogenic impact, the home range of brown bears is also influenced by food availability [14,15,16] as well as by the interaction with conspecifics [17,18]. Brown bears are not specialized regarding their diet [19,20] and, therefore, show a high ecological flexibility [21,22]. Still, their behavior strongly depends on the season, which leads to specific food and nutrition demand, respectively [16,23]. During autumn, which precedes denning, bears show hyperphagic behavior and need a rich food availability to gain weight [16]. Hyperphagia can thus be considered the most critical time of year for brown bears as they might face stress through their dietary needs as well as through potential human–bear conflicts [10,15]. Bears seem to prefer natural food sources over artificial feeding sites as this might increase the risk of a human encounter [15]. Despite being an omnivorous generalist that consumes a wide range of plants and animals [24], 76–80% of the brown bear diet consists only of plant material and 20–24% includes both plant and animal material, while the consumed animals were mostly insects [25,26]. In particular, ants play an important role in the diet of brown bears as they provide a high nutritional value [27,28]. During autumn, bears’ main food resources are fruits and beechnuts [26,29] and they additionally consume ants [30]. However, artificial feeding sites can influence the movements and home range size, especially when natural food resources are poor [25,26,31]. It has been observed that bears more frequently visit artificial feeding sites in years with low mast production; conversely, in years with good mast production, they were not frequent at feeding sites [25]. When foraging, bears exploit the available food source, as scat and stomach analyses showed that a major part of samples contained only one or two food items [25,26]. In the case of plentiful food supplies, home ranges tend to be smaller because the animal can forage on spatially dense food resources [32,33,34]. During hyperphagia and before the hibernation period begins in autumn, home range size often changes as diet directly influences the home range of brown bears [31,35,36].

The aim of this study is to understand the home range and space use of two female brown bears during the most critical phase of their phenology, especially as both females were gravid during this period. This study is the first to investigate movements of brown bears in the Velebit Mountains in Croatia [16,31,34] considering the reproductive state. It shows the importance of areas without human presence inside a National Park that is frequently visited by tourists. Thus, conservation efforts can be adapted and management of tourism and other wildlife affecting actions in this human dominated landscape can be improved.

## 2. Materials and Methods

### 2.1. Study Area and Study Species

**Figure 1:**
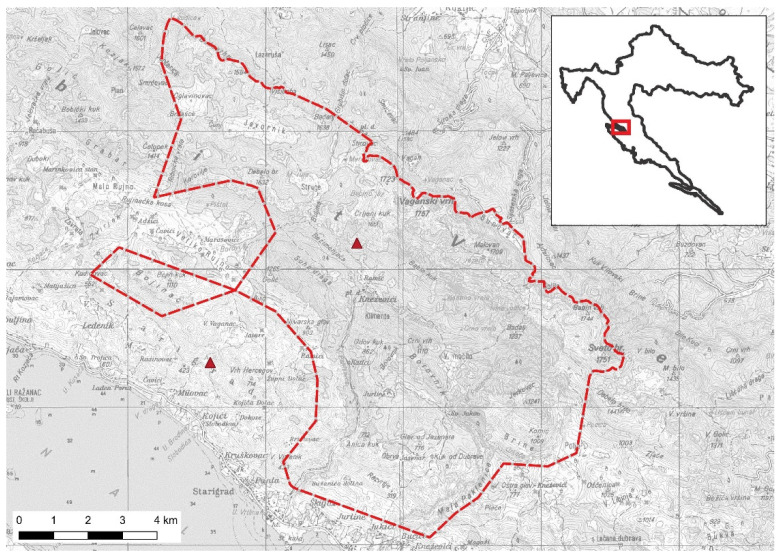
Paklenica National Park is located close to the Adriatic coast northeast of Starigrad, Croatia. Red dashed line shows the border of the National Park; red triangles show denning sites of the two female bears. Scale: 1:75,000. Small map (top right) shows the location of the National Park (red) in Croatia.

We conducted our study in Paklenica National Park and its surrounding areas (Figure 1). The park is characterized by a climatic gradient from Mediterranean to Continental and Alpine climate throughout the change in altitude from 0 m to 1757 m above sea level [37,38]. The varying climate as well as the height difference shaped a heterogenous environment with oak (*Quercus pubescens*) forest in the lower altitudes, beech forests (*Fagus sylvatica*) in higher altitude and *Fagetum subalpinum* and *Pinetum mugi* forest in the highest altitudes [39]. In the different habitat types, different natural food resources can be found such as nuts (beech nuts (*Fagus sylvatica*), acorns (*Quercus spec.*)) and fruits (cherries (*Cornus mas*), blackberries (*Rubus fructicosus*), figs (*Ficus spec.*)). Various species of ants can be found in the whole National Park [40].

From spring until autumn, the park attracts a great number of tourists for hiking and rock climbing in the great canyon of the National Park. In 2019, a total of 144,680 tourists visited the National Park [39]. The park is closely located to the city of Starigrad (<1900 inhabitants) [41] and other smaller towns. There are also smaller settlements and single houses close to the outside border of the park as well as inside the park. On the plateau Veliko Rujno, close to the western border of the National Park, smaller settlements are inhabited all year round. A smaller group of permanently used settlements is in the center of the park—the Ramić area. Some of these houses are used as dorms for tourists. Another three dorms are distributed throughout the whole park site as well as a few more single houses that are used on the weekends or by farmers that have their cattle inside the National Park. None of the settlements have more than ten households [42].

The brown bear population in Croatia is estimated at 937 individuals (range 846–1072) with the highest density in the Gorski Kotar region and northern and middle Velebit mountain with about 1.5–2 individuals per 10 km^2^ [6,43]. The sex ratio is 42:58 in favor of females [43]. Hunting and supplemental feeding of brown bears is legal outside of National Park borders (EU Habitat Directive article 16, Nature Protection Act articles 85 and 151 and Croatian Hunting Act articles 3 and 60). For each hunting ground, owners of hunting rights must determine and register feeding sites for bears annually. Feeding is allowed only in years in which the hunting right owners have permission to hunt bears and only during hunting seasons [6]. In addition to the registered bear feeding sites, there are numerous feeding sites for wild boars and deer that are also visited by bears throughout the year [15].

### 2.2. Capturing of Bears

We live captured two female brown bears at the same location, Njivarski bunar (44°20′03.0″ N, 15°27′29.0″ E), inside the National Park on the 10 and 15 September 2019. For capturing, we used Aldrich spring-activated foot snares. We established temporary feeding sites prior to capturing, which we supplied with bait (fish remains, fruit, vegetables and bakery products) to lure the bears to the trapping site. We equipped each trapping site with a GSM alarm system, which notified us if the animal was in the trap. As soon as possible after the alarm notification, the National Park team checked the trap. We tranquilized the captured bears using an injection gun (DAN-INJECT ApS, Børkop, Denmark) with a mixture of tiletamine hydrochloride and zolazepam hydrochloride (Zoletil, Virbac, Carros, France) and medetomidine hydrochloride (Domitor, Vetoquinol, Towcester UK). Once immobilized, we measured the bears and sampled according to standard protocol. We determined the sex of the animals and approximate age. Later, their age was determined by counting the cementum annuli in teeth, which was performed by Matson’s Laboratory LLC, Manhattan, MT, USA [44]. We fitted each bear after being measured and sampled with the GPS/GSM collar, which also includes VHF/UHF communication (VECTRONIC Aerospace GmbH, Berlin, Germany). Animal capture and handling procedures were approved by the Croatian Ministry of Nature Protection and Energetics and the Ministry of Agriculture, since brown bears in Croatia are a strictly protected species according to the EU and national law (Council Directive 92/43/EEC on the conservation of natural habitats and of wild fauna and flora—Habitat Directive and Nature Protection Act, respectively) and are listed as game animals according to the Croatian Hunting Act.

### 2.3. Estimation of Home Range, Overlapping Territories, Proximity to Feeding Sites and Settlements

In autumn of 2019, we collected GPS data of two adult female brown bears (individuals “B95” and “B97”) for approximately two months during their hyperphagic stage and before the denning period. We used GPS data for analyses from the date of capture until the individuals started denning on 4 and 14 November 2019, respectively. We set the collection of GPS locations by the collar on every hour. The monthly success of recorded GPS positions was, on average, 96.6% for bear B95 and 98.7% for bear B97. We eliminated GPS positions with errors (e.g., latitude and/or longitude were not recorded). In total, we analyzed 96.3% and 90.1%, respectively, of the recorded data and a total of 2733 GPS positions for both females. We applied the Brownian Bridge Movement Model (BBMM) to estimate home ranges because it gives a more reasonable range in comparison to classical home range estimators, it is constructed on the properties of a conditional random walk between recorded locations and it accounts for spatiotemporal correlation between successive relocations [45]. In this way, it allows for a more accurate view on the spatial use of an animal than other approaches [45]. We delimited bear home ranges using the 95% isopleth of the utilization distribution estimated with the Brownian Bridge Movement Model. We performed BBMM following Calenge (2006) [46] calculating σ1 (Brownian motion variance) using the maximum likelihood approach (R function liker in adehabitatHR package). We estimated σ2 (location imprecision) as ±25 m as GPS location error [47]. We calculated BBMM in R statistics (R version 3.6.3, 29 February 2020) using the adehabitat package [46]. As the two bears ranged in a mountainous landscape, we corrected the total surface of home ranges taking into consideration the difference in elevation between movement locations. For this purpose, we used the 3D analyst tool of ArcGIS Pro (version 2.8.0) with the European Digital Elevation Model (EU-DEM, version 1.0) with a 25 m resolution, provided by the European Environment Agency (EEA) under the framework of the Copernicus programme [48,49]. Following De Angelis et al., 2021 [31], we provided minimum convex polygon (MCP) as 95% of the most outer recorded GPS fixes for comparison with earlier studies. To analyze the spatial-temporal overlap between the two individuals, we calculated the utilization distribution overlap index (UDOI) for every month (September–November) in the adehabitatHR package in R. The UDOI value ranges between 1 (complete overlap) and 0 (no overlap), but might also be greater than 1 if distributions are non-uniformly distributed and extensively overlapping [50]. We chose the UDOI for our analysis as this allows for continuous spatial utilization distribution estimated with BBMM, likely being more informative than other indices [50].

For analyzing the potential human impact, the National Park team provided information about the location of settlements and artificial feeding sites. We analyzed the potential use of the artificial feeding site by identifying the GPS locations of the bears in proximity of less than 1000 m distance to the feeding sites, as we assumed that bears could clearly reach this distance within one hour. We then calculated the percentage of time spent in this proximity. For calculating the distance from each GPS fix of a bear to the closest settlement, we used distance matrix in QGIS (version madeira 3.4.13). We tested differences in the distances to the closest settlements between the months using the Kruskal–Wallis test and multiple comparison test after Kruskal–Wallis as a post hoc test. In order to reveal activity patterns during the hyperphagic period, we have calculated the Euclidean distance between consecutive GPS fixes of the two bears [51]. We consider movements of less than 25 m as inactive according to the GPS location error [47]. For all statistical analyses and plots, we used R statistics (version 3.6.3 29 February 2020). We set the significance level for all tests to α = 0.05.

## 3. Results

### 3.1. Home Range

During the hyperphagic period, the seasonal home range of individual B95 was greater compared to B97 in September, but smaller in October (Table 1). Mean home ranges with standard deviation (±SD) for the months of September and October 2019 were 5.6 ± 0.4 km^2^ for bear B95 and 5.6 ± 1.2 km^2^ for bear B97. Bear B95 spent 90.7% of its time outside of the National Park and only 9.3% inside. Bear B97 spent 39.5% inside and 60.5% outside the National Park. 

### 3.2. Overlapping Territories

**Figure 2:**
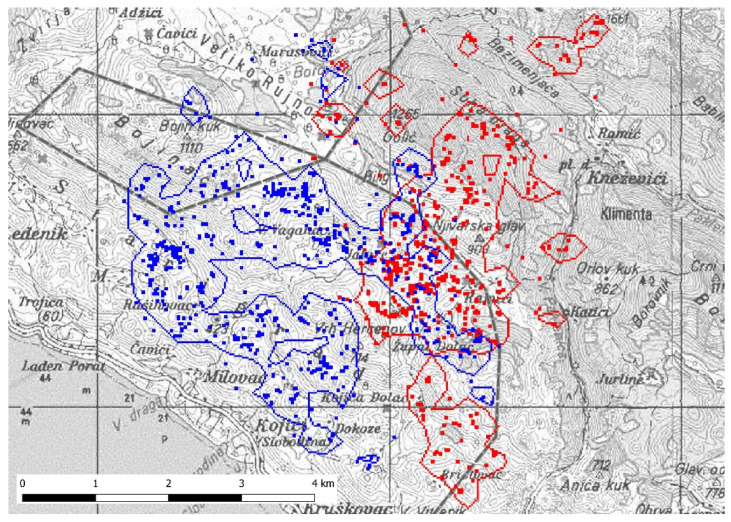
Seasonal home ranges (BBMM 95%) of bear B95 (blue) and bear B97 (red) for September until November 2019. Dark grey dashed line shows National Park border. Scale: 1:33,000.

In September 2019, both bears shared an area of 1.5 km^2^ with a UDOI of 0.08 (Figure 2). In October, they shared a total area of 0.2 km^2^ with a UDOI of 0.001 and, in November, they did not share habitat (UDOI = 0).

### 3.3. Proximity to Feeding Sites and Settlements

**Figure 3:**
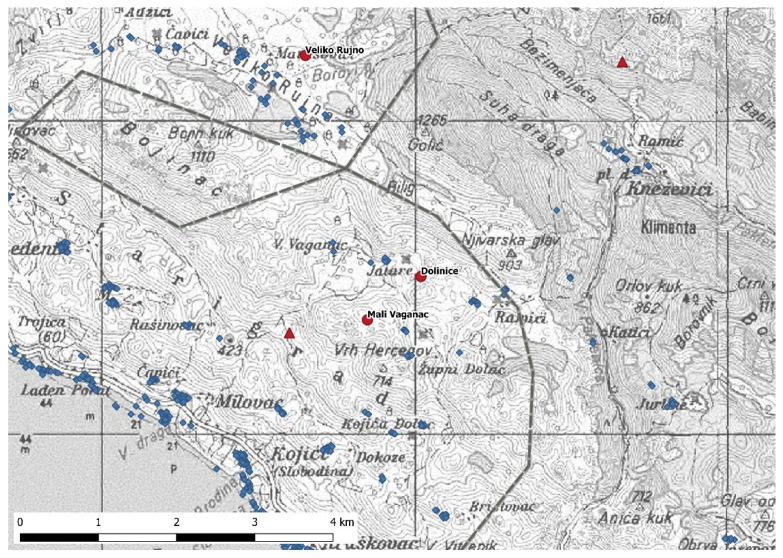
The three artificial feeding sites (red dots) are located close to the border of the National Park (dark grey dashed line) and to settlements (blue squares). Red triangles show denning sites of the two bears. Scale: 1:33,000.

Three artificial feeding sites can be found close to the border of Paklenica National Park (Figure 3). The site Veliko Rujno is located on the high plateau of the same name. Next to this site are several settlements, while only few houses are found close to the other two locations (Figure 3). Bear B95 spent about 40% and 38% of its time close to the artificial feeding sites (i.e., <1000 m) in September and October, respectively. In November, it came close to the site Mali Vaganac and spent only about 16% of the time in proximity to it. Bear B97 spent about 45% in September and about 93% in October close to the feeding sites. In November, it spent about 76% of its time close to Dolinice and about 32% close to Mali Vaganac. The last two feeding sites are located less than 1000 m away from each other (Figure 3).

The closest distance from the GPS fixes to the settlement measured for bear B95 between September and November 2019 was 8.9 m, while the greatest distance was 1678.9 m. For bear B97, the closest distance was 4.4 m and the greatest 2027.9 m. They quite frequently approached houses, for instance, in the settlements on Veliko Rujno where a feeding site is also located. Bear B95 used a den site on approximately 570 m AMSL while bear B97′s den was located higher on circa 1100 m AMSL (Figure 3). Den sites were 895.4 and 1071.1 m away from the closest settlement for bear B95 and B97, respectively. For both bears, we found no significant difference in the distance to the closest settlements between October 2019 and November 2019 (Kruskal–Wallis test, *p* > 0.05), whereas the difference was significant between September 2019 and October 2019 as well as between September 2019 and November 2019 (Kruskal–Wallis test, *p* < 0.05). For both individuals, the distance to settlements was closest right before denning in November (Table 2).

**Figure 4:**
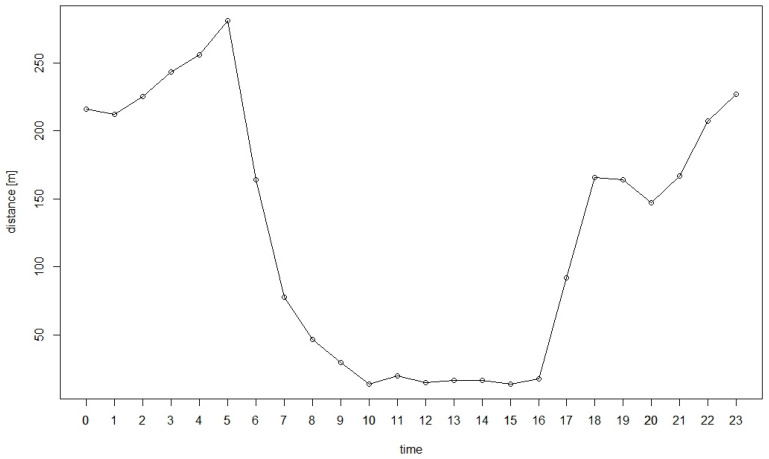
Mean distance (m) travelled hourly of the two bears during day and night in the hyperphagic period. Time shows the hours of the day.

The analysis of the activity pattern showed that the bears were more active during the night (Figure 4). They become active at 4 o’clock in the afternoon and reach their movement activity peak between 5 and 6 o’clock in the morning. At 10 o’clock, they become inactive. 

## 4. Discussion

Our study was the first to provide insight into the seasonal home range size of two gravid female brown bears in Paklenica National Park and surroundings. We analyzed the hyperphagic behavior of two individuals by studying their home range, the spatiotemporal overlap and their proximity to human settlements and artificial feeding sites. We found that the home ranges of the two studied gravid female brown bears were very small during hyperphagia with values of 9.3 km^2^ and 7.5 km^2^, respectively (BBMM 95% for September–November 2019). Home range sizes of male and female brown bears in Croatia have only recently been studied, showing that average home ranges (BBMM 95%) in autumn during hyperphagia are smallest at about 55 km^2^ [31]. Seasonality, age, supplemental feeding site density and time of day are known factors for influencing the home range of brown bears [26,31,32]. Other studies showed that male bears have larger home ranges than females, which is, among other considerations, due to factors associated with their larger body size [52,53]. Furthermore, female brown bears can remain in an exceedingly small area. Seryodkin et al., 2012 reported a brown bear female with cubs to use an area as little as 0.2 km^2^ for seven consecutive days (Kernel 95%) [54]. McLoughlin et al., 2000 have compared several studies about female brown bear home ranges (MCP) in North America [32]. The mean annual home range was 376.6 km^2^, while the smallest reported annual home range was 24 km^2^ and the largest 2577 km^2^. Annual mean home ranges (MCP) of 28 km^2^ for female brown bears are known from previous studies conducted in Croatia, while some subadult females have annual home ranges as small as 7.9 km^2^ [55]. The estimated seasonal home ranges of the two gravid females in our study are even smaller in comparison to the smallest home ranges reported from other studies of adult females. As the present study was conducted in Paklenica National Park and its surroundings, the strong gradient in altitude creates a very heterogenous habitat for brown bears. Mangipane et al., 2017 stated that brown bears use smaller home ranges in heterogenous habitats. This can be one explanation for the small home ranges of the brown bears found in this study. Furthermore, UDOI indicated only very little to no simultaneously used area by bears. Often, territoriality is rendered unnecessary if food availability is high, especially in female brown bears [56,57]. Our findings, however, suggest that at least these two females use distinct areas in the pre-denning stage as they showed almost no overlapping habitat use. Additionally, female brown bears are even known to demonstrate territorial behavior towards other females as well as reported cases of intraspecific attacks and even infanticide [58,59]. The fact that both females were gravid during hyperphagia [35,60] might be one additional explanation for the small seasonal home range size. Gravid females start denning earlier than males or females with offspring [35,36]. Therefore, they may keep their home ranges as small as possible to avoid encounters with conspecifics to not lose their unborn. In white-tailed deer (*Odocoileus virginianus*), gravidity had a major impact on the movement of females as the home range of non-gravid females without fawns was more than 50% larger in comparison to females that were gravid and females that had fawns (within season and year) [61]. To our knowledge, the influence of gravidity of female brown bears on the home range size has not been studied, and further research should be conducted in this field. Nevertheless, our results suggest that bears may need enough space for solitary habitat use within their home range. Moreover, it can be assumed that food resources were high as this leads to a decreased movement of the bears during hyperphagia [32,33,34]. However, this remains an assumption as food abundance was not yet analyzed during this period in the Velebit Mountains. Both bears spent a considerable amount of time close to artificial feeding. A recent dietary study about brown bears in Croatia stated that bears use artificial feeding sites more often in poor mast years [25]. We can assume that frequent use of artificial feeding sites increased the amount of available food resources, hence decreased movement and home ranges for these two females. Further studies should therefore be conducted on the diet and influence of supplemental feeding on home ranges in Paklenica National Park. Female brown bears generally avoid human presence if possible [9,62]. However, this study revealed that while moving within their home ranges, bears passed by very closely to human settlements. Mean distance to human settlements during months preceding the denning period even became lower. Furthermore, the bears are mostly active during night, which is consistent within the literature, as a way to avoid interactions with humans [8]. If shelter in the habitat is offered through structures that are inaccessible to humans, female brown bears can use areas close to anthropogenic infrastructures [9]. This is applicable for the heterogenous landscape of the Velebit Mountains with the strong altitude differences and several caves and structures to hide in. Even though human infrastructure and presence can be tolerated by brown bears during their hyperphagic period, it nevertheless remains very critical as human bear encounters occur most often during hyperphagia and the bears are active in the early morning and late afternoon [10]. In autumn, there are still many tourists inside the National Park [39]. In addition, human disturbance might have a harmful impact on the bears during the denning period, which follows hyperphagia [16]. To avoid this problem, bears should be offered enough space and it is advisable to establish feeding sites further away from human settlements, which is also regulated by law accordingly as feeding sites need to be at least 2 km away from settlements [6].

## 5. Conclusions

This study was the first to analyze the home ranges of two gravid female brown bears in Paklenica National Park and its surrounding area during hyperphagia, revealing very small seasonal home ranges during this season. Yet, the brown bears used the space almost exclusively showing only little overlap. To avoid human–bear conflict and to fulfil the bears’ spatial requirements, bears need concealed areas during hyperphagia with little to no human disturbance. Further studies should be conducted in Paklenica National Park as this study only provided data about movements of two gravid female brown bears and only during one season. Therefore, more individuals of different age, sex, and reproductive category should be collared and tracked to support the findings of this study and help improve management and conservation efforts. Moreover, the question about the actual diet of the brown bears as well as the potential influence of gravidity on home range remains unclear and should be answered in future studies.

## Figures and Tables

**Table 1:** Brownian Bridge Movement Model (BBMM) in km^2^ of bear B95 and B97 from September until November 2019 as 95% of their seasonal home range as well as minimum convex polygon (MCP) as 95% in km^2^. In November, bear B95 was active for 14 days and bear B97 for only four days.

		September 2019		October 2019		November 2019		September–November 2019	
Bear ID	Age (years)	BBMM (95%)	MCP (95%)	BBMM (95%)	MCP (95%)	BBMM (95%)	MCP (95%)	BBMM (95%)	MCP (95%)
B95	5	6.0	9.0	5.4	8.2	2.7	3.7	9.3	16.3
B97	7	4.8	11.1	6.6	12.6	0.2	0.2	7.5	14.5

**Table 2:** Mean distance ± SD (m) between each GPS fix and closest settlement for the two bears B95 and B97 from September 2019 until November 2019.

Bear ID	September 2019	October 2019	November 2019
B95	723.0 ± 370.3	568.3 ± 282.7	534.4 ± 223.6
B97	760.8 ± 400.1	570.3 ± 326.0	562.5 ± 163.0

## Data Availability

Restrictions apply to the availability of these data. Data was obtained from National Park Paklenica and are available from the corresponding author with the permission of National Park Paklenica.
